# Kelulut Honey Regulates Sex Steroid Receptors in a Polycystic Ovary Syndrome Rat Model

**DOI:** 10.3390/ijms232314757

**Published:** 2022-11-25

**Authors:** Datu Agasi Mohd Kamal, Siti Fatimah Ibrahim, Azizah Ugusman, Mohd Helmy Mokhtar

**Affiliations:** 1 Department of Physiology, Faculty of Medicine, Universiti Kebangsaan Malaysia, Kuala Lumpur 56000, Malaysia; 2 Department of Biomedical Sciences, Faculty of Medicine and Health Sciences, University Malaysia Sabah, Kota Kinabalu 88400, Malaysia

**Keywords:** honey, PCOS, oestrogen receptors, androgen receptor, progesterone receptor

## Abstract

Reproductive and metabolic anomalies in polycystic ovary syndrome (PCOS) have been associated with the dysregulation of sex steroid receptors. Kelulut honey (KH) has been shown to be beneficial in PCOS-induced rats by regulating folliculogenesis and the oestrus cycle. However, no study has been conducted to evaluate KH’s effect on sex steroid receptors in PCOS. Therefore, the current study examined the effects of KH, metformin, or clomiphene alone and in combination on the mRNA expression and protein distribution of androgen receptor (AR), oestrogen receptor α (ERα), oestrogen receptor β (ERβ), and progesterone receptor (PR) in PCOS-induced rats. The study used female Sprague-Dawley rats, which were treated orally with 1 mg/kg/day of letrozole for 21 days to develop PCOS. PCOS-induced rats were then divided and treated orally for 35 days with KH, metformin, clomiphene, KH + metformin, KH+ clomiphene and distilled water. In this study, we observed aberrant AR, ERα, ERβ and PR expression in PCOS-induced rats compared with the normal control rats. The effects of KH treatment were comparable with clomiphene and metformin in normalizing the expression of AR, ERα, and ERβ mRNA. However, KH, clomiphene and metformin did not affect PR mRNA expression and protein distribution. Hence, this study confirms the aberrant expression of sex steroid receptors in PCOS and demonstrates that KH treatment could normalise the sex steroid receptors profile. The findings provide a basis for future clinical trials to utilize KH as a regulator of sex steroid receptors in patients with PCOS.

## 1. Introduction

Polycystic ovary syndrome (PCOS) is a combination of endocrine, reproductive, and metabolic disorders characterised by aberrant folliculogenesis, anovulation, irregular menstrual cycles, insulin resistance, and hyperandrogenism [1,2]. PCOS is the most prevalent hormonal disorder in reproductive-age women [3,4]. However, due to its heterogeneous characteristics, reports on its prevalence have varied, with stricter diagnostic criteria resulting in a prevalence of 6%. In comparison, broader diagnostic inclusion criteria increase the prevalence to 20% among reproductive-age women [5,6].

To date, the etiopathogenesis of PCOS is still not well defined [7,8]. However, the suggested etiopathology of PCOS includes abnormalities in steroidogenesis [9,10], follicular arrest [11], deficiency in aromatase enzymes [12], and insulin-resistant hyperinsulinism [13]. Additionally, recent studies have investigated the role of sex steroid receptors in women with PCOS [14,15,16]. Studies on the expression of these receptors and the associated abnormalities have triggered a new understanding of PCOS aetiology [17].

Sex steroid receptors, including oestrogen receptor (ER), androgen receptor (AR), and progesterone receptor (PR), are physiologically limited not only to the female reproductive system, but are also crucial for the non-reproductive functions such as the cardiovascular and metabolic systems [18]. A recent study discovered that the dysregulation of sex steroid receptors is the cause of aberrant follicular development and metabolic diseases, such as obesity, diabetes mellitus, cardiovascular disease, and hypertension in PCOS [17]. Meanwhile, another study discovered ER- and AR-mediated mechanisms responsible for generating various PCOS phenotypes [19]. Studies on the differential expression of sex steroid receptors have proven that these receptors are aberrantly expressed in PCOS [14,16,17].

Because the definitive aetiology of PCOS is not yet finalised, treatment goals mainly stipulate reducing the symptoms. Several drugs currently being utilised include clomiphene for ovulation induction, oral contraceptives to regulate the menstrual cycle, and metformin to manage insulin resistance. However, some reports have shown that these medications are associated with adverse effects, including gastrointestinal upset, abdominal pain, and uterine bleeding [20,21]. In addition, approximately 40% of women with PCOS were reported to experience clomiphene resistance, which further limits the treatment choice for patients [22]. According to a study, 70% of Australian women with PCOS are using complementary treatments, and adverse side-effects were reported in 12.2% of respondents [23]. Thus, exploring effective and safe nutraceuticals such as honey is essential to providing complementary treatment options for PCOS patients.

Kelulut honey (KH) is a natural stingless bee honey reported to be effective in ameliorating both female and male reproductive system disorders [24,25]. KH possesses excellent antioxidative, anti-inflammatory, anti-cancer, and anti-diabetic properties [26]. Previously, we have proved that KH could improve oxidative stress, hormonal imbalance, folliculogenesis, and steroidogenic and aromatase enzyme profiles in PCOS-induced rats [27,28]. Thus, the current study is an extension of our previous investigations, aiming to discover the effects of KH on sex steroid receptors in PCOS rats. The effects of KH, metformin, or clomiphene on AR, ERα, ERβ, and PR profiles were compared and discussed.

## 2. Results

### 2.1. Effect of KH on AR mRNA Expression and Protein Distribution

**Figure 1:**
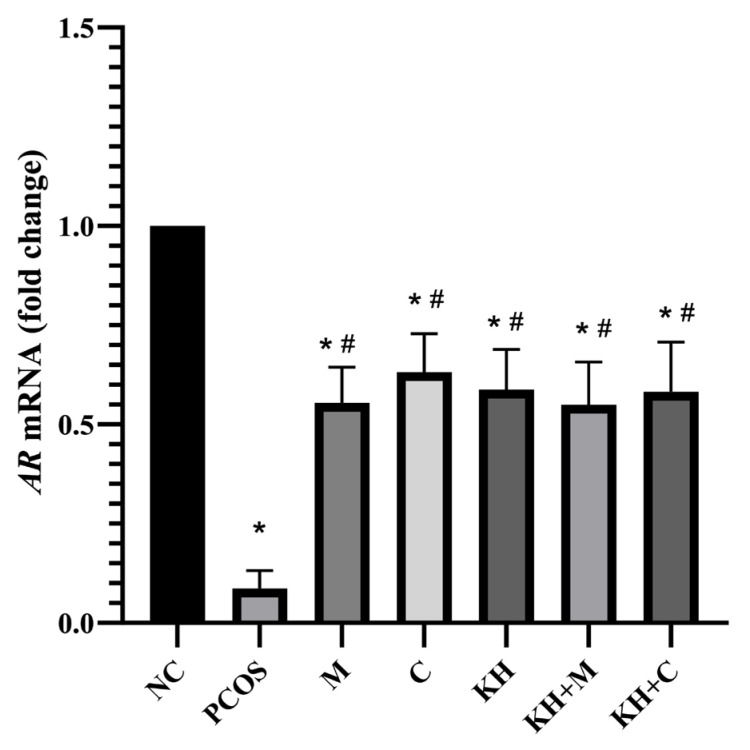
Effects of KH on AR mRNA expression. NC: normal control; PCOS: untreated PCOS; M: PCOS + metformin; C: PCOS + clomiphene; KH: PCOS + 1 g/kg/day of Kelulut honey; KH + M: PCOS + 1 g/kg/day of Kelulut honey + metformin; KH + C: PCOS + 1 g/kg/day of Kelulut honey + clomiphene. * *p* < 0.05 significance compared to the normal control group, # *p* < 0.05 significance compared to the untreated PCOS group, *n* = 6 per treatment group.

Figure 1 demonstrates the effect of KH on AR mRNA expression. AR mRNA expression was significantly downregulated in the untreated PCOS group compared with the normal control rats (0.08 ± 0.01 vs. 1.00 ± 0.00, *p* < 0.05). The downregulation of the AR gene was significantly (*p* < 0.05) improved to near normal levels following treatment with KH (0.59 ± 0.04), clomiphene (0.63 ± 0.04), combined KH + clomiphene (0.58 ± 0.05), combined KH + metformin (0.55 ± 0.04), and metformin (0.55 ± 0.04). However, there were no significant differences observed between the treatment groups.

**Figure 2:**
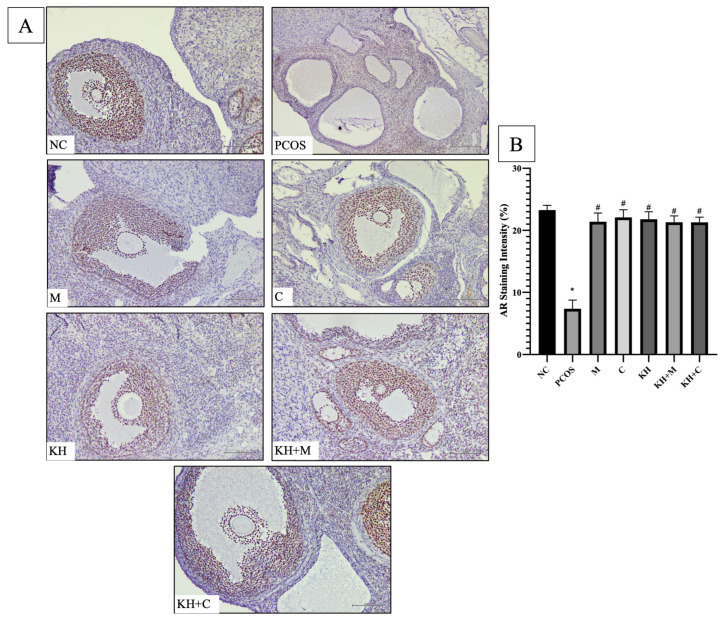
(**A**) Representative images of AR protein distribution and (**B**) quantitative analysis of AR protein staining intensity in the treatment groups. The antibody-binding site of AR is marked by the dark brown stain present in granulosa cell nuclei of primary follicles, antral follicles and preovulatory follicles. NC: normal control; PCOS: untreated PCOS; M: PCOS + metformin; C: PCOS + clomiphene; KH: PCOS + 1 g/kg/day of Kelulut honey; KH + M: PCOS + 1 g/kg/day of Kelulut honey + metformin; KH + C: PCOS + 1 g/kg/day of Kelulut honey + clomiphene. Scale bar = 100 μm. Magnification 200× and 400×. * *p* < 0.05 significance compared to the normal control group, # *p* < 0.05 significance compared to the untreated PCOS group. *n* = 6 per group.

Meanwhile, Figure 2A shows the distribution of AR protein in rat ovaries. Positive DAB staining was localised at the granulosa cell nuclei of primary, antral, and preovulatory follicles in all groups. In addition, in every group, staining was absent in cystic follicles and the corpus luteum. As shown in Figure 2B, staining intensity was significantly reduced in untreated PCOS rats compared with normal control rats (7.36 ± 0.57% vs. 23.27 ± 0.31%, *p* < 0.05). This reduction was significantly (*p* < 0.05) improved to near normal levels following treatment with clomiphene alone (22.09 ± 0.50%), KH + clomiphene (21.30 ± 0.33%), KH alone (21.8 ± 0.49%), metformin (21.39 ± 0.57%) and KH + metformin (21.30 ± 0.42%). There were no significant differences observed between the treatment groups.

### 2.2. Effect of KH on ERα and ERβ mRNA Expression and Protein Distribution

**Figure 3:**
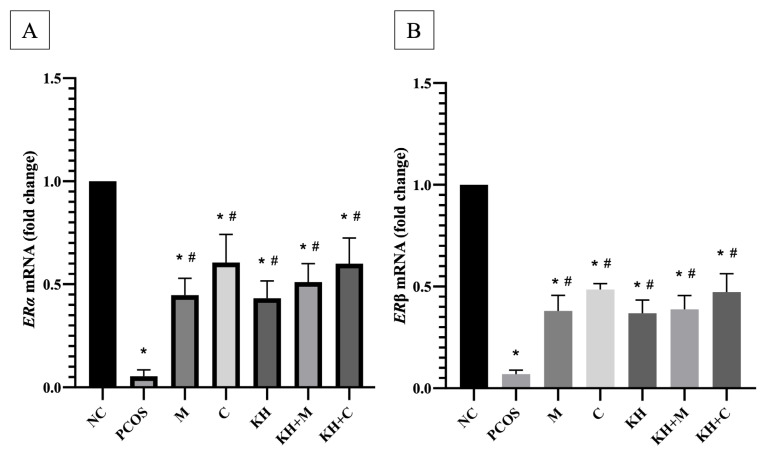
Effects of KH on (**A**) ERα and (**B**) ERβ mRNA expression. NC: normal control; PCOS: untreated PCOS; M: PCOS + metformin; C: PCOS + clomiphene; KH: PCOS + 1 g/kg/day of Kelulut honey; KH + M: PCOS + 1 g/kg/day of Kelulut honey + metformin; KH + C: PCOS + 1 g/kg/day of Kelulut honey + clomiphene. * *p* < 0.05 significance compared to the normal control group, # *p* < 0.05 significance compared to the untreated PCOS group, *n* = 6 per treatment group.

Figure 3A shows the effects of KH on ERα mRNA expression. PCOS induction caused significant downregulation of the ERα mRNA expression in untreated PCOS rats compared with the normal control group (0.05 ± 0.01 vs. 1.00 ± 0, *p* < 0.05). Treatment with clomiphene (0.6 ± 0.06), combined KH + clomiphene (0.6 ± 0.05), combined KH + metformin (0.51 ± 0.04), metformin (0.45 ± 0.04), and KH (0.43 ± 0.03) significantly increased ERα mRNA expression (*p* < 0.05) compared with the untreated PCOS rats (0.05 ± 0.01). However, no significant differences were recorded among the treatment groups.

ERβ mRNA expression is shown in Figure 3B. PCOS induction caused ERβ mRNA expression to be downregulated significantly (*p* < 0.05) in untreated PCOS rats compared with the normal control rats (0.07 ± 0.01 vs. 1.00 ± 0, *p* < 0.05). Treatment with clomiphene (0.49 ± 0.03), KH + clomiphene (0.47 ± 0.04), KH + metformin (0.39 ± 0.03), metformin (0.38 ± 0.03), and KH only (0.37 ± 0.03) significantly upregulated ERβ mRNA expression (*p* < 0.05) compared with the untreated PCOS group (0.07 ± 0.01). There were no significant differences observed between the treatment groups.

**Figure 4:**
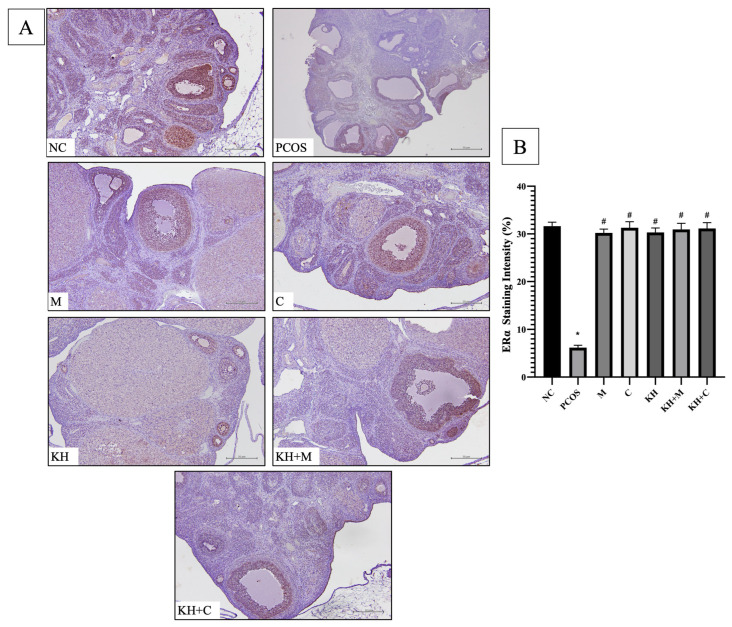
(**A**) Representative images of ERα protein distribution and (**B**) quantitative analysis of ERα protein staining intensity in the treatment groups. The antibody-binding site of ERα is marked by the dark brown stain that is present in granulosa cells and theca cells of primary follicles, antral follicles, and preovulatory follicles. NC: normal control; PCOS: untreated PCOS; M: PCOS +metformin; C: PCOS + clomiphene; KH: PCOS + 1 g/kg/day of Kelulut honey; KH + M: PCOS + 1 g/kg/day of Kelulut honey + metformin; KH + C: PCOS + 1 g/kg/day of Kelulut honey + clomiphene. Scale bar = 50 μm. Magnification 100×, *n* = 6 per group. * *p* < 0.05 significance compared to the normal control group, # *p* < 0.05 significance compared to the untreated PCOS group. *n* = 6 per group.

Figure 4A demonstrates the ERα protein distribution. In all groups, DAB staining was found in the granulosa and theca cells of primary, antral, and preovulatory follicles. In addition, in every group, staining was absent in cystic follicles and the corpus luteum. The staining intensity of ERα (Figure 4B) was significantly decreased in untreated PCOS rats compared with normal control rats (31.63 ± 0.34% vs. 6.16 ± 0.21%, *p* < 0.05). This reduction was significantly reversed to a near normal level (*p* < 0.05) by treatment with KH + clomiphene (31.14 ± 0.49%), clomiphene alone (31.29 ± 0.52%), KH alone (30.31 ± 0.38%), KH + metformin (30.94 ± 0.53%), and metformin (30.21 ± 0.33%). There were no significant differences observed between the treatment groups.

**Figure 5:**
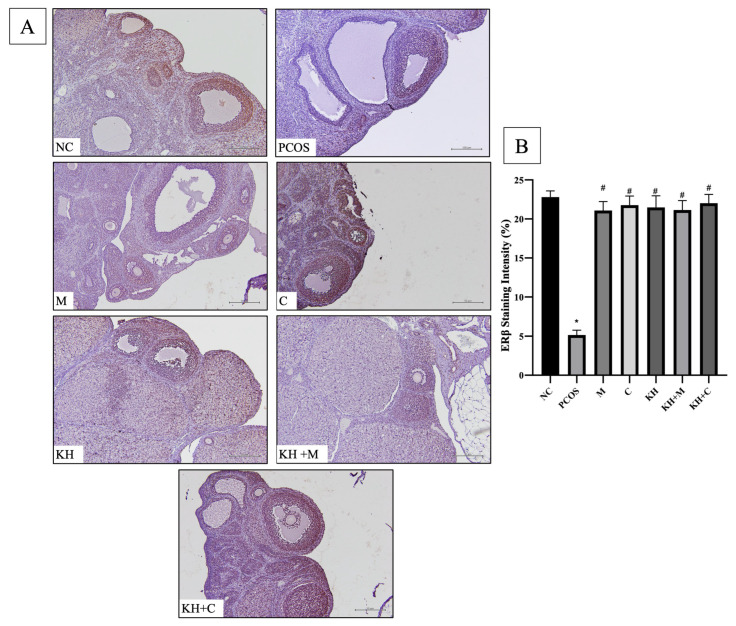
(**A**) Representative images of ERβ protein distribution and (**B**) quantitative analysis of ERβ protein staining intensity in the treatment groups. The antibody-binding site of ERβ is marked by the dark brown stain present in the granulosa cells and theca cells of primary follicles, antral follicles, and preovulatory follicles. NC: normal control; PCOS: untreated PCOS; M: PCOS + metformin; C: PCOS + clomiphene; KH: PCOS + 1 g/kg/day of Kelulut honey; KH + M: PCOS + 1 g/kg/day of Kelulut honey + metformin; KH + C: PCOS + 1 g/kg/day of Kelulut honey + clomiphene. Scale bar = 50 μm and 100 μm. Magnification 100×, *n* = 6 per group. * *p* < 0.05 significance compared to the normal control group, # *p* < 0.05 significance compared to the untreated PCOS group. *n* = 6 per group.

The distribution of ERβ protein in rat ovaries is shown in Figure 5A. Positive DAB staining was localised in the granulosa and theca cells of primary follicles, antral follicles, and preovulatory follicles in all groups. In addition, in every group, staining was absent in the cystic follicles and corpus luteum. As shown in Figure 5B, the staining intensity of ERβ was significantly decreased in untreated PCOS rats compared with normal control rats (22.80 ± 0.32% vs. 5.15 ± 0.25%, *p* < 0.05). This reduction was significantly reversed to near normal (*p* < 0.05) by treatment with KH + clomiphene (22.02 ± 0.46%), clomiphene alone (21.77 ± 0.48%), KH alone (21.48 ± 0.61%), KH + metformin (21.14 ± 0.49%), and metformin (21.07 ± 0.47%). There were no significant differences observed between the treatment groups.

### 2.3. Effect of KH on PR mRNA Expression and Protein Distribution

**Figure 6:**
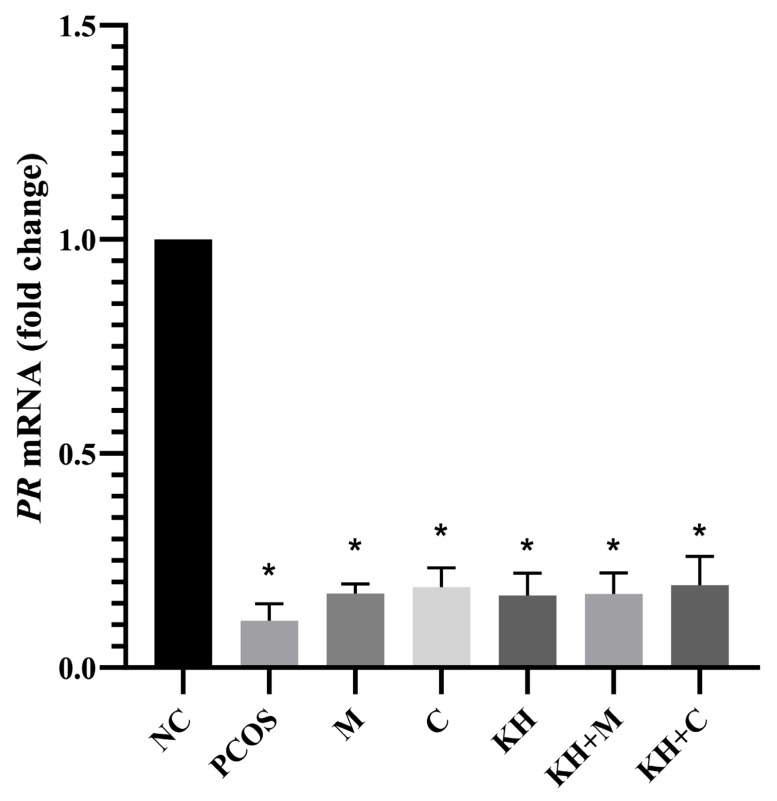
Effects of KH on mRNA expression of PR. NC: normal control; PCOS: untreated PCOS; M: PCOS + metformin; C: PCOS + clomiphene; KH: PCOS + 1 g/kg/day of Kelulut honey; KH + M: PCOS + 1 g/kg/day of Kelulut honey + metformin; KH + C: PCOS + 1 g/kg/day of Kelulut honey + clomiphene. * *p* < 0.05 significance compared to the normal control group, *n* = 6 per treatment group.

**Figure 7:**
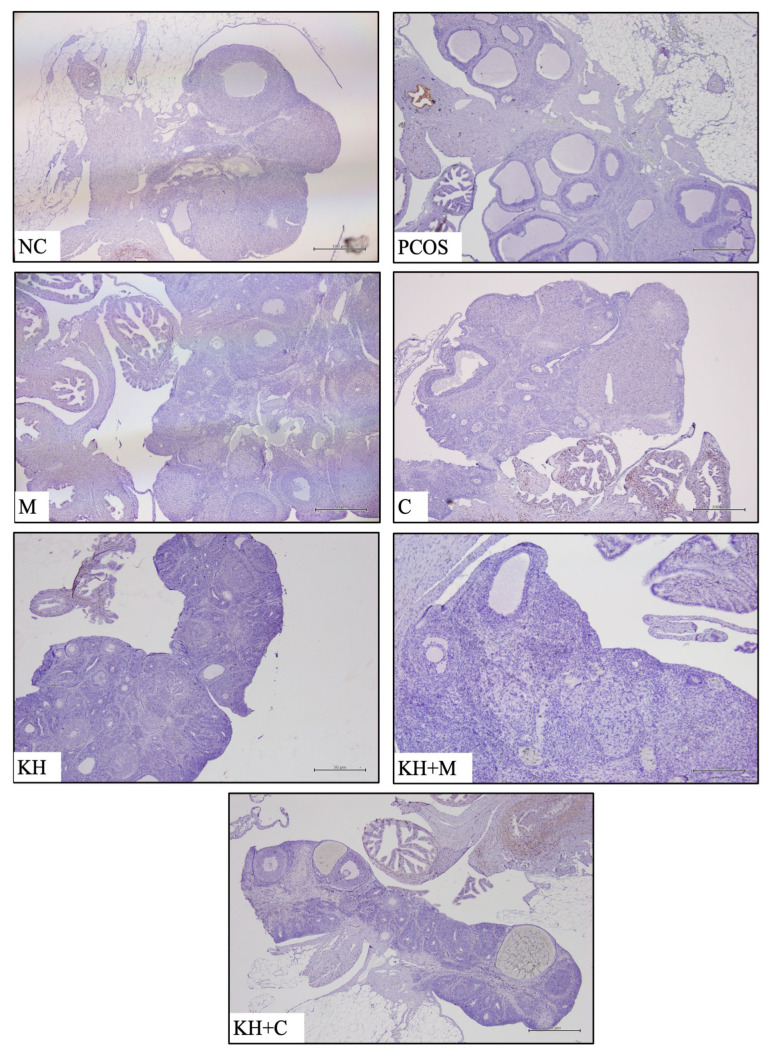
Effect of KH on PR protein distribution. The antibody-binding site of PR is marked by the dark brown stain that presents only in the oviduct and not in the ovary. NC: normal control; PCOS: untreated PCOS; M: PCOS + metformin; C: PCOS + clomiphene; KH: PCOS + 1 g/kg/day of Kelulut honey; KH + M: PCOS + 1 g/kg/day of Kelulut honey + metformin; KH + C: PCOS + 1 g/kg/day of Kelulut honey + clomiphene. Scale bar = 50 μm and 100 μm. Magnification 40×, *n* = 6 per group.

As shown in Figure 6, the mRNA expression of PR was significantly downregulated in untreated PCOS rats compared with the normal control rats (0.11 ± 0.02 vs. 1.00 ± 0.00, *p* < 0.05). However, the treatment groups did not differ from the untreated PCOS group. Meanwhile, Figure 7 demonstrates the PR protein distribution. In all groups, DAB staining was not found in the whole ovary, but positive staining was recorded in the oviduct.

## 3. Discussion

According to Paris et al., hyperandrogenism, working via AR, is the key player in the pathogenesis of PCOS [29]. Hyperandrogenism causes abnormal folliculogenesis, insulin resistance, aromatase enzyme deficiency, and physical disturbances such as hirsutism and androgenic alopecia in PCOS patients [30,31]. A previous study demonstrated that blocking AR gene expression in female mice can prevent PCOS-like pathology development, indicating the role for AR in PCOS pathogenesis [32]. Furthermore, several studies have reported that the mRNA expression of AR in ovarian granulosa cells of women with PCOS was significantly lower than in normal healthy women [14,31,33]. In this study, we recorded downregulation of AR mRNA expression in PCOS rat ovaries compared with normal rats. However, it is unclear whether AR expression is upregulated [34,35] or downregulated [36] in the ovaries of PCOS-induced animals. Gao et al. explained the discrepancy in AR expression in human and animal studies. They proposed that short-term stimulation of androgen will increase AR expression in vitro or in vivo, followed by inhibition of AR expression due to the increased AR-mediated reaction in the chronic hyperandrogenism of PCOS. This AR downregulation hinders follicle development, which causes PCOS patients’ ovaries to produce many arrested antral follicles [14]. Nonetheless, whether it is over- or under-expressed, abnormal expression of AR is always associated with abnormal folliculogenesis in PCOS [17].

We also demonstrated that KH was comparable with clomiphene and metformin in reversing the downregulation of AR mRNA expression. Previously, a review article concluded that honey could significantly regulate testosterone levels [37] which may indirectly show its action on the AR. In vitro studies have shown that caffeic acid phenethyl ester, the major constituent of honey bee propolis and KH [38,39], regulates AR via inhibiting phosphorylation of serine 81 and serine 213 [40]. Previously, we demonstrated that KH could normalise elevated serum testosterone and LH levels with improved oxidative stress status in PCOS rats [28]. Additionally, treatment with KH reduced the elevated expression of Cyp17a1 (a steroidogenic enzyme) in rats with PCOS [27]. An interplay between testosterone, LH, steroidogenic enzymes, and oxidative stress status may be the possible pathway that mediates the KH effect on AR expression. Previous studies also have highlighted trehalulose, a bioactive disaccharide and phenolic content of stingless bee honey or KH, for its active effects mainly achieved via the antioxidant pathway [41,42]. The anti-oxidative pathway of KH may be one of the possible mechanisms underlying its effect on the sex steroid receptors. Meanwhile, another study revealed that *Ecklonia cava*, a plant with excellent anti-oxidative properties [43], significantly reversed AR mRNA downregulation in letrozole-induced PCOS rats [36].

In this study, clomiphene, metformin, and a combination of the drugs with KH were shown to reverse the effects of PCOS induction on AR. Furthermore, metformin therapy in PCOS reduces hyperandrogenaemia, facilitating normal menses and pregnancy [44]. A previous study showed that metformin could downregulate AR expression in the endometrium of PCOS patients [45]. Meanwhile, clomiphene has been shown to significantly increase the mRNA expression of AR in the testes of lead acetate-induced rats [46]. AR protein was reported to be localised in the nuclei of granulosa cells in primordial, primary, and secondary follicles [35]. In line with the previous study, our findings showed AR to be positively stained at the granulosa cell nuclei of primary, antral, and preovulatory follicles in all the groups. The untreated PCOS group recorded significantly lower staining intensity than the normal control rats.

Oestrogen mediates its effect through three types of receptors; the genomic pathway via ERα and ERβ, and the non-genomic pathway via the G-protein-coupled oestrogen receptor (GPER). However, Barton et al. [47] found that GPER has a lesser binding affinity for oestrogen compared with ERα and ERβ receptors. All three oestrogen receptors are encoded by a unique gene on separate chromosomes [48]. *Esr1* and *Esr2* encode the ERα and ERβ receptors, respectively. [16]. The importance of both oestrogen receptors was proven when ERα knock-out mice demonstrated the features of ovarian cysts, haemorrhagic follicles, elevated androgen levels, and irregular oestrus cycles [49]. Meanwhile, ERβ knock-out mice showed the characteristics of upregulated steroidogenic enzyme expression, formation of cystic follicles, and elevated LH levels [50]. PCOS has been linked to aberrant expression of both ERα and ERβ, and this causes folliculogenesis and ovulatory failure [16,51]. In this study, we found that ERα and ERβ mRNA expression levels were significantly downregulated in the group of untreated PCOS rats compared with the normal control group. This is in line with other studies, which reported that letrozole-induced PCOS rats have downregulation of ERα [52] and ERβ mRNA expression [36]. In addition, mice with disrupted ERα genes were characterised by elevated LH concentrations, haemorrhagic ovaries, cystic follicles, and failure of ovulation, resembling PCOS conditions [53,54]. Meanwhile, ERβ knockout mice developed aberrant folliculogenesis, decreased ovulation, and fewer pregnancies [55,56]. It has been reported that ER is crucial in maintaining normal folliculogenesis, ovulation, and ovarian granulosa cell development [57].

We found that treatment with KH significantly reversed the downregulation of both ERα and ERβ mRNA expression. Previously, various honeys, including Tualang and Manuka honey have been reported to modulate ER activity through their phenolic content [58,59]. This may be achieved as the phenolic ring is reported to be able to bind into both ERα and ERβ [60]. Furthermore, according to Ahmed et al., Tualang and Manuka honey may alter the dimerization of ER through nuclear cytoplasmic shuttling modification hence blocking the ER nuclear localization [59]. KH phenolic content might be a related agent, evidenced by its effect on modulating ERα and ERβ in this study. In fact, resveratrol, a natural phenolic compound, is reported to be an agonist to the ER [61]. It has been shown that the concentration of honey determines its oestrogenic or anti-oestrogenic effects [62,63,64]. However, our previous study shows KH does not affect the estradiol level in PCOS-induced rats [28]. While we recorded upregulation of ER with KH treatment, other studies have reported that Tualang and Manuka honey downregulate ER expression [59,65]. This discrepancy warrants future investigation for validation. In addition, the KH effect on ERα and ERβ was comparable with clomiphene, metformin, or a combination of either drug with KH. Clomiphene is well known as a selective ER modulator [66,67]. Clomiphene works by facilitating folliculogenesis and increasing the ovulation rate [68]. The binding of clomiphene to the hypothalamic ER stimulates the release of gonadotropins from the anterior pituitary, which eventually promotes the recruitment of healthy antral follicles for ovulation [69]. Meanwhile, metformin is the first-line drug to manage diabetes in PCOS patients. In addition to controlling diabetes, a study revealed that metformin improved the ovulation rate in PCOS patients [70]. Metformin has also been reported to enhance folliculogenesis in PCOS-induced rats [71], while an in vitro study demonstrated that metformin could modulate both ERα and ERβ [72]. In this study, we found ERα and ERβ to be positively stained in granulosa and theca cells of primary, preantral, and antral follicles. In healthy women, ER is expressed in granulosa and theca cells in developing follicles [16]. In adult rodents, ERα is predominantly found in the theca cells, and ERβ in the granulosa cells of developing follicles [73]. However, Yang et al. reported staining location overlaps between ERα and ERβ in developing follicles in rodents [74].

Progesterone has been shown to act primarily on the endometrium via PR, with essential roles in endometrial receptivity, embryo implantation, placental development, and parturition [75]. Furthermore, animal study has proven PR to be crucial in ovulation. For example, a study found that PR knock-out mice will develop ovulation failure, even in response to exogenous hormones [76]. In addition, the granulosa cells of women with PCOS had lower mRNA expression of PRA and PRB than healthy women [77]. In this study, we found that letrozole induction downregulated PR mRNA expression. Previously, Lee et al. reported similar findings where letrozole-induced PCOS rats showed lower PR mRNA expression than controls [52].

However, we found that treatment with KH, clomiphene, metformin, or a combination of them did not cause any changes to PR mRNA expression. Similarly, our previous finding demonstrated the KH did not alter progesterone levels in PCOS-induced rats. Metformin has been reported to regulate progesterone levels in women with PCOS [78]. In PCOS-induced rats, metformin reverses the decreased progesterone level caused by PCOS induction [79], whereas clomiphene treatment has a variable effects on decreased progesterone levels in PCOS-induced rats. Ndeingang et al. [79] found no difference, while Atashpour et al. [80] recorded a significant reversal in progesterone levels. Previous studies in animals have demonstrated that Tualang honey and Gelam honey can regulate progesterone levels [81,82]. However, further study on the effects of KH, metformin, and clomiphene on PR is needed to validate their effects and to enrich the limited data available.

Our study revealed the absence of PR staining in the ovaries of all groups, although the oviduct and endometrium were well stained. It has been reported that PR protein is chiefly expressed in the endometrium and oviduct [83]. Previous studies have documented the physiological absence of PR staining in the corpus luteum of normal rats [84,85,86]. Although the onset of PR expression in rat follicles is rapid, it is decreased several hours after an LH surge and becomes undetectable once the corpus luteum forms [83,85,87]. Thus, PR is expressed only in the growing ovarian follicles observed later in the proestrus phase in response to the ovulatory surge of pituitary gonadotrophins, strengthening its role in ovulation and luteinisation [84]. Furthermore, ovarian follicles in pregnant rats were also reported not to be immunostained for PR [84]. As discussed, the rats in this study were sacrificed during the dioestrus phase, which may be the reason for the absence of PR-positive staining. However, further investigation is required to explain the differential expression of PR, particularly in women with PCOS.

## 4. Materials and Methods

### 4.1. Honey Sample

Kelulut honey from *Heterotrigona itama* stingless bees originated from Negeri Sembilan, Malaysia; the bees feed on various local herbal plants. The honey was collected in the middle of April 2021. It was kept unprocessed and refrigerated at 4 °C in amber bottles covered from heat sources and sunlight.

### 4.2. Ethics and Animals Information

The study was conducted on healthy female Sprague–Dawley rats (120–150 g) with a minimum of two consecutive regular oestrus cycles. The rats were acquired from the Laboratory Animal Research Unit, Faculty of Medicine, National University of Malaysia. They were acclimatized for a week and maintained at 24 ± 2 °C with a 12 h light/12 h dark cycle. Animals were fed with standard food pellets and provided with water ad libitum. The study was approved by the National University of Malaysia Animal Ethics Committee with Ethical registration number FISIO/FP/2020/MOHD HELMY/14-MAY/1104-JUNE-2020-MAY-2023.

### 4.3. Study Design

**Figure 8:**
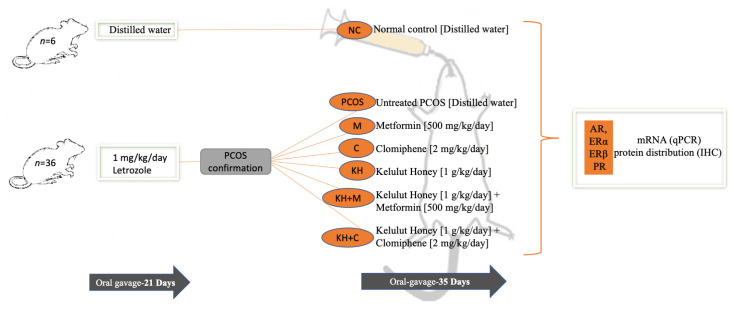
The illustration depicts the study design. AR: androgen receptor; ER: oestrogen receptor; PR: progesterone receptor; qPCR: quantitative polymerase chain receptor; IHC: immunohistochemistry.

Forty-two female rats were divided into two major groups, as shown in Figure 8. The first group (*n* = 36) was the PCOS induction group, treated with 1 mg/kg/day of letrozole by oral gavage for 21 days. Meanwhile, the second group (*n* = 6) was given distilled water during the study (56 days). The PCOS induction method was taken from a validated study described by Kafali et al. [88]. The PCOS rats were shown to develop irregular oestrous cycles, hyperglycaemia, and ovarian cysts [25].

After the rats were confirmed to have developed PCOS, they were randomly distributed into six groups with six rats per group: rats receiving 1 g/kg/day KH; 500 mg/kg/day metformin; 2 mg/kg/day clomiphene; 500 mg/kg/day metformin + 1 g/kg/day KH; 2 mg/kg/day clomiphene + 1 g/kg/day KH; and distilled water. All treatments were administered by oral route for 35 days. KH dose (1 g/kg/day) and treatment duration (35 days) were determined from our pilot study [25]. The 2 mg/kg/day clomiphene and 500 mg/kg/day metformin effective doses were obtained from a previous investigation by Ndeingang et al. [79]. Metformin is a recommended treatment for insulin resistance, whereas clomiphene stimulates ovulation in PCOS patients [89]. The groups receiving metformin and clomiphene were designated positive controls to assess the effects of KH treatment. Additionally, a combination of clomiphene and metformin with KH was intended to evaluate any synergistic effect. Finally, the animals were sacrificed by the ketamine–xylazine overdose method, administered intraperitoneally at a dose of 0.3 mL/100 g rat body weight [90].

### 4.4. Sex Steroid Receptor Distribution Analysis by Immunohistochemistry (IHC)

After sacrificing the animals, the right ovaries were excised, cleaned from fat, and preserved in a 10% neutral buffered formalin solution at 4 °C. These tissues were then processed and embedded in paraffin wax. Then, tissues were cut into sections of 5 μm thickness and fished into poly-lysine-coated slides. To improve the antigen–antibody binding, slides were treated with an antigen retrieval solution (Dako, Glostrup, Denmark) pH 9.0 or 6.0 in a decloaking chamber (Biocare Medical, Pacheco, CA, USA). A micro-polymer mouse- and rabbit-specific HRP/DAB IHC detection kit (Abcam, Cambridge, MA, USA) was used as per the manufacturing guidelines. Briefly, slides were treated with hydrogen peroxide to prevent endogenous peroxidase and incubated with blocking serum. Next, the slides were treated with rabbit polyclonal antibodies against androgen receptor (AR) (Cat AB133273 Abcam) in 1:300 dilution, oestrogen receptor α (ERα) (Cat AB3575 Abcam) in 1:400 dilution, oestrogen receptor β (ERβ) (Cat AB5786 Abcam) in 1:200 dilution, and progesterone receptor (PR) (Cat AB16661 Abcam) in 1:400 dilution. The slides were then treated with a micro-polymer secondary antibody. To visualise the protein, the slides were treated with DAB substrate. Finally, sections were sequentially stained with haematoxylin and dehydrated in alcohol. The slides were visualised using an Olympus BX40 light microscope (Olympus Corporation, Tokyo, Japan). The positive control tissues were rat testis tissue (for AR), human breast tissue (for ERα), rat testis tissue (for ERβ), and rat placental tissue (for PR). DAB staining in the obtained images was analyzed using the ImageJ Fiji software (version 1.2; WS Rasband, National Institute of Health, Bethesda, Rockville, MD, USA) based on the validated protocol [91].

### 4.5. Sex Steroid Receptor Gene Expression Analysis by Real-Time PCR

Left ovaries were cut into smaller pieces and immersed in RNA Later solution (Sigma Aldrich, Saint Louis, MO, USA) to keep the RNA in the tissues intact. Then, ovarian tissue lysates were powdered using a mortar and pestle in liquid nitrogen. Following the manufacturer’s protocols, RNA was extracted using the Nucleospin RNA isolation kit (Macherey-Nagel, Duren, Germany). The absorbance of every extraction was determined at 260 nm and 280 nm, and RNA quality was assessed by the 260/280 ratio (Gene Quant 1300, Cambridge, UK). Reverse mRNA transcription was conducted using the OneScript^®^ Hot cDNA synthesis kit (Applied Biological Materials (ABM) Inc., Vancouver, BC, Canada). Transcriptase-free amplification of samples was used as an experimental control. The qPCR master mix used is BlasTaq 2X qPCR MasterMix (Applied Biological Materials (ABM) Inc., Vancouver, BC, Canada). Glyceraldehyde-3-phosphate dehydrogenase (GAPDH) was used as a housekeeping gene.

Validated specific primers were obtained from Sigma Aldrich, Saint Louis, MO, USA and are depicted in Table 1. Real-time quantitative PCR was performed using the BioRad CFX96 real-time system. The machine was set as follows: 3 min at 95 °C to activate the polymerase enzyme, followed by 15 s at 95 °C for 40 cycles for the denaturation step, then1 min at 60 °C for 40 cycles for annealing and elongation. The experiment was carried out in triplicate. Data were analyzed according to the comparative CT (2^−ΔΔCt^) method. The relative expression quantity of each amplicon was measured by comparing the normalized expression quantity of each gene to the normalised expression quantity of the reference gene.

### 4.6. Statistical Analysis

Data analysis was carried out using GraphPad Software version 8 (GraphPad Inc., San Diego, CA, USA). Statistical analysis was conducted using one-way ANOVA, followed by Tukey’s multiple comparison tests to determine differences among groups. Data were expressed as mean ± SEM. *p* values of less than 0.05 were set as statistically significant.

## 5. Conclusions

This study demonstrates that sex steroid receptors are abnormally expressed in PCOS-induced rats. Treatment with KH could normalise the mRNA expression and protein distribution of AR, Erα, and Erβ, and these effects of KH are comparable with those of clomiphene and metformin. This investigation provides a foundation for future clinical trials to utilize KH as a regulator of sex steroid receptors in PCOS patients.

## Figures and Tables

**Table 1:** The sequence of specific primers used.

Target Genes	Forward (F) and Reverse (R) Primer Sequence
*AR*	F CCTTGTTCCCTTTTCAGATG R GTAAAAGAGGCAGAGAAGAAG
*ESR1*	F ATATGATCAACTGGGCAAAG R CATTTACCTTGATTCCTGTCC
*ESR2*	F GGAAATCTTTGACATGCTCC R GGTACATACTGGAGTTGAGG
*PGR*	F TCTAATCCTGAATGAGCAGAG R GACTTTCATACAGAGGAACTC
*GAPDH*	F CTCAATGGGAACTTAACAGG R CTCTGTATAAGCAAGGATGC

*AR*: androgen receptor gene; *ESR1*: oestrogen receptor α gene; *ESR2*: oestrogen receptor β gene; *PGR*: progesterone receptor gene; *GAPDH*: GAPDH gene.

## Data Availability

The data is contained within the article.
